# Impact of High-Fiber or High-Protein Diet on the Capacity of Human Gut Microbiota To Produce Tryptophan Catabolites

**DOI:** 10.1021/acs.jafc.2c08953

**Published:** 2023-05-01

**Authors:** Zhan Huang, Jos Boekhorst, Vincenzo Fogliano, Edoardo Capuano, Jerry M. Wells

**Affiliations:** † Food Quality and Design Group, Department of Agrotechnology and Food Sciences, Wageningen University and Research, P.O. Box 17, 6700 AA Wageningen, The Netherlands; ‡ Host-Microbe Interactomics Group, Department of Animal Sciences, Wageningen University and Research, P.O. Box 17, 6700 AA Wageningen, The Netherlands

**Keywords:** diet, SHIME, gut microbiota, tryptophan, indole derivatives

## Abstract

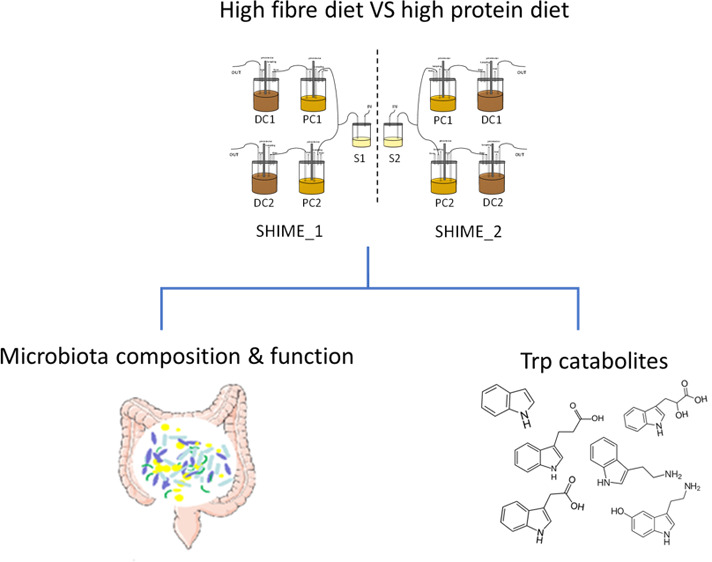

This study investigated the effect of high-fiber-low-protein (HF) and high-protein-low-fiber (HP) diets on microbial catabolism of tryptophan in the proximal colon (PC) and distal colon(DC) compartments of the Simulator of the Human Intestinal Microbial Ecosystem. The microbiota in PC and DC was dominated by Bacteroidetes and Firmicutes, in which Bacteroidetes were more abundant in DC (∼60% versus 50%) and Firmicutes were more abundant in PC (∼40% versus 25%). Most of the tryptophan catabolites were determined at a higher concentration in PC samples than in DC samples, but the overall concentration of tryptophan catabolites was over 10-fold higher in DC samples than that in PC samples. Interestingly, indole-3-propionic acid and oxindole were only identified in DC samples. A two-week dietary intervention by the HF diet enriched the abundance of Firmicutes in PC, whereas the HP diet enriched the abundance of Proteobacteria. Compared to the HP diet, the HF diet favored the microbial production of indole-3-acetic acid, indole-3-lactic acid, indole-3-aldehyde, and indole-3-propionic acid in both PC and DC compartments. To conclude, these findings increase the understanding of the effect of diets on the microbial production of tryptophan catabolites in the colon.

## Introduction

There is a growing interest in manipulating the gut microbiota and microbiota-derived metabolites though diet to beneficially modulate host physiology.^1−4^ The most studied microbial metabolites are short-chain fatty acids (SCFAs) mainly produced by the colonic fermentation of undigested complex polysaccharides.^5^ SCFAs have profound effects on gut homeostasis, exert anti-inflammatory and epigenetic effects, and affect physiology in other organs through G-protein-coupled receptor signaling.^6−8^ Recently, indole derivatives originating from microbial catabolism of tryptophan (Trp) have received much attention, due to their aryl hydrocarbon receptor (AhR)-dependent anti-inflammatory effects and modulation of innate lymphoid cells type 3, which produce IL-22,^9,10^ a cytokine important for intestinal repair and barrier function.^11^ Several studies have reported the beneficial effects of indole derivatives on host physiology.^12−15^ Individuals with metabolic syndrome,^15^ inflammatory bowel disease,^16^ and celiac disease,^17^ have a reduced concentration of indole derivatives in the feces, presumably due to the altered gut microbial community. A large human cohort study on the correlation between circulating concentrations of Trp metabolites and the incidence type 2 diabetes (T2D) showed that indole-3-propionic acid (IPA) was inversely associated with T2D risk.^18^ Recently, IPA was shown to promote regeneration and functional recovery of nerves after injury.^19^ It is important to notice that Trp metabolism via the indolic pathway by gut microbiota also produces some uremic toxins, such as indole-3-acetic acid (IAA) and indoxyl sulfate, that exert a deleterious effect on multiple organs in patients with chronic kidney disease.^20^

Several *Bacteroides* spp., *Clostridium* spp., *Bifidobacterium* spp., *Lactobacillus* spp., and *Peptostreptococcus* spp. have been reported to be able to convert Trp into indole derivatives,^10,13,21,22^ but the complete set of taxa and the related microbial pathways are incomplete. Emerging evidence from animal and human studies suggests a potential role of diet in the manipulation of Trp catabolism by gut microbiota.^23−25^ The fecal concentration of microbiota-derived Trp catabolites was increased in mice and pigs fed with a high-Trp diet.^17,26^ A recent study showed that human intake of fiber-rich foods was positively associated with the serum concentrations of IPA,^18^ a finding confirmed in older adults on a polyphenol-rich diet.^27^ Previously, using an *in vitro* batch model of colonic fermentation, we demonstrated that dietary fibers (i.e., pectin and inulin) are able to promote the microbial production of IPA, IAA, and indole-3-lactic acid (ILA).^28^ However, the effects of long-term dietary habits on the microbial production of Trp catabolites in the intestine have not yet been systematically explored.

Here, we tested the effects of two different diets, a high-fiber-low-protein (HF) diet and a high-protein-low-fiber (HP) diet, on the microbial production of Trp catabolites in the proximal and distal colon using the simulator of human intestinal microbiota ecosystem (SHIME), a dynamic gastrointestinal model for long-term microbiome intervention studies.^29^ SHIME was inoculated with *ex vivo* human gut microbiota to mimic the microbiological properties of the proximal and distal parts of the colon under mimicking in vivo conditions. To determine the effects of diet on the functional capacity of the microbiota, as well as microbiota composition and diversity, we used 16S rRNA amplicon sequencing and shotgun metagenomic sequencing. In addition, we also quantified a panel of microbiota-derived Trp catabolites in the proximal colon (PC) and distal colon (DC) compartments of SHIME and correlated their concentration with the abundance of different taxa at each location to provide insights into the main producers of Trp catabolites in the human colon.

## Materials and Methods

### Ethics

The Medical Ethical Committee of East Netherlands declared that this study does not fall under the Medical Research Involving Human Subjects Act (WMO), which requires assessment by the METC of the East Netherlands or another recognized medical-ethical review committee. The subjects that provided a fecal sample for SHIME were deemed not to be subject to acts or any conduct that is subject to the Medical Research Involving Human Subjects Act.

### Experimental Design

**Figure 1:**
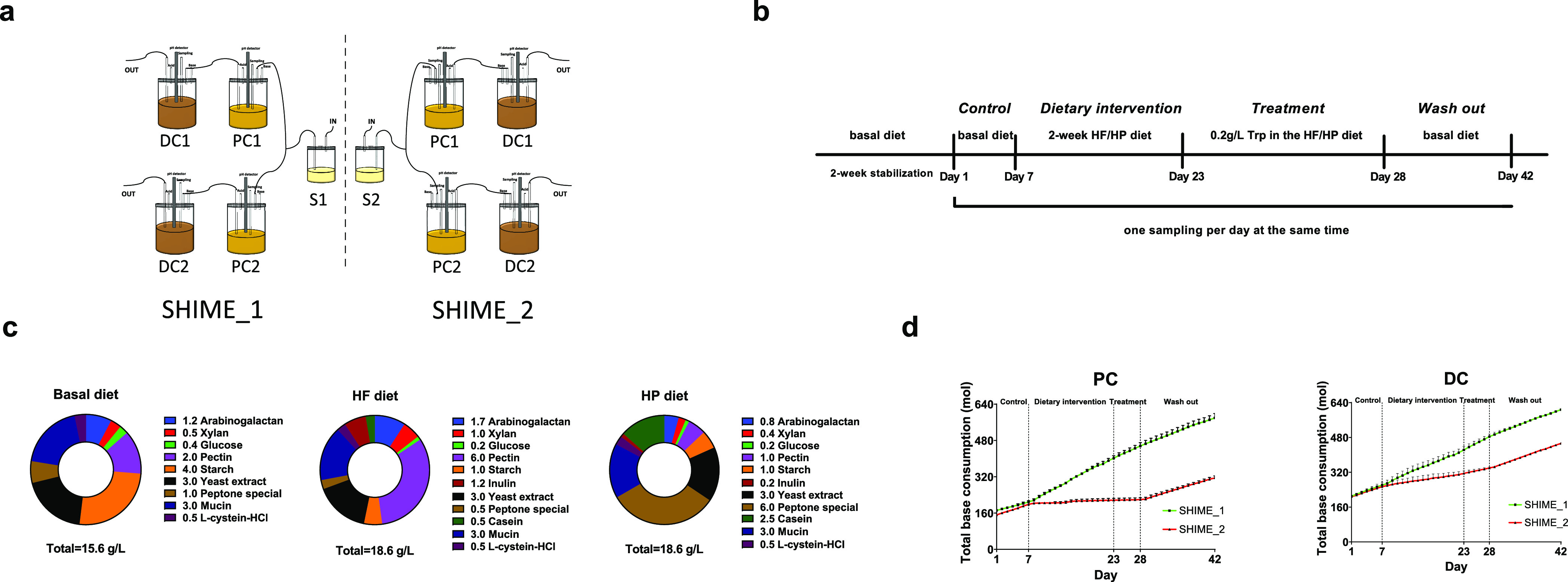
Overview of the study design. (a) The design of the SHIME model mimicking the proximal colon (PC) and distal colon(DC). (b) A schematic of the experimental procedure including two-week microbiome stabilization, one-week control period, two-week dietary interventions, one-week tryptophan (Trp) treatment, and two-week wash-out. (c) The composition of the basal, high-fiber-low-protein (HF), and high-protein-low-fiber (HP) diets for SHIME microbiota. (d) The total consumption of the base solution of the SHIME model during the study. Control: SHIME fed with basal diet to measure the baseline. Dietary intervention: SHIME_1 fed with high-fiber-low-protein (HF) diet and SHIME_2 fed with high-protein-low-fiber (HP) diet. Treatment: supplied 0.2 g/L tryptophan in the HF/HP diet. Wash out: SHIME fed with basal diet. The data are presented as mean + SD (*n* = two biological donors).

SHIME (ProDigest, Belgium) was set up as previously described.^30^ Briefly, as shown in Figure 1a, SHIME consisted of two units (SHIME_1 and SHIME_2). Each unit had one combined stomach and small intestine vessel (S1/S2) and was subdivided into two parallel PC and DC compartments, which were inoculated with fresh fecal samples from two healthy donors. As host genetics, sex, age, diet, and drugs affect the gut microbiota composition and functionality,^31,32^ the selected donors were both Dutch females, aged 22 to 25, with a normal BMI value, similar dietary habits, and no history of antibiotic and probiotic use in the last 6 months prior to donation, to reduce inter-individual differences in gut microbiome. The SHIME system was kept at 37 °C by means of a warm water circulator (AC200, Thermo Fisher Scientific). The pH was maintained at 5.6–5.9 for PC compartments and at 6.6–6.9 for DC compartments by adding 0.5 M HCL (acid) or NaOH (base). In the rest of the manuscript, microbiota adapted to the PC or DC compartment of SHIME are referred to as PC or DC microbiota, respectively. The feeding was programmed three times per day with an interval of eight hours. In each cycle, 224 mL of fresh feed (pH 1.8–2.2) was pumped to S1/S2 and later was mixed with 96 mL of pancreatic juice (12.5 g/L NaHCO_3_, 6 g/L Oxgall, and 0.9 g/L pancreatin) to simulate the digestion happening in the small intestine. After 90 min, the digesta was parallelly transferred to the series of PC and DC compartments within 60 min. The volume of PC and DC compartments was kept constant at 400 and 640 mL, respectively, all the time.

The experiment was conducted in five stages, as illustrated in Figure 1b. To adapt to the new environment and produce a representative microbial community, the *ex vivo* human gut microbiota was stabilized for two weeks by continuously feeding with the adult SHIME growth medium (basal diet) (PD-NM001B, ProDigest). A control period supplied with the basal diet for one week was placed to measure the baseline (days 1–7). Next the dietary intervention was given for two weeks (days 8–23), in which the SHIME_1 unit was supplied with a HF diet and the SHIME_2 unit with a HP diet. To verify the diet effect on microbial production of Trp catabolites, an extra intervention referred to as “treatment” was performed by supplementation of 0.2 g/L free Trp in the HF or HP diet for five days (days 24–28). Lastly, the basal diet was used for a wash-out period of two weeks (days 29–42) to determine whether there were lasting effects of the different diets on the microbiota. The details of the diet composition are reported in Figure 1c. All ingredients were purchased from ProDigest (Gent, Belgium), except inulin (product No. I2255) and casein (product No. C8654) from Sigma-Aldrich (St. Louis MO, USA). HF or HP diet was formulated by changing the composition and amounts of the ingredients in the basal diet, essentially as previously described.^33^ The total consumption of base solution as an indicator of the fermentation process is shown in Figure 1d, which was automatically added to neutralize the SCFAs produced in the PC and DC compartments of SHIME so as to maintain the pH range. Samples from the fermenter were collected once per day at the same time starting point from the control period, and they were immediately centrifuged after sampling (12,500 rpm, 5 min, 4 °C). The supernatants were filtered using a 0.2 μm regenerated cellulose filter (Phenomenex, Torrance, CA) and stored at −20 °C until the Trp metabolites were measured. The pellets were kept at −80 °C for extraction of bacterial DNA and sequencing.

### LC–MS Analysis of Trp Catabolites

Trp catabolites in the supernatant were quantified as previously described.^28,34^ Briefly, the centrifuged and filtered supernatants of fermented samples were diluted 10-fold with Milli-Q water before being subjected to targeted analysis for Trp catabolites, including IPA, IAA, ILA, indole (Ind), oxindole (Oxi), indoleacrylic acid (IA), indole-3-aldehyde (I3A), tryptamine (TA), kynurenine (Kyn), and serotonin (5-HT) were measured via a Shimadzu Nexera XR LC-20ADxr UPLC system coupled with a Shimadzu LCMS-8050 mass spectrometer (Kyoto, Japan). Chromatographic separation was accomplished on a Phenomenex Kinetex 1.7 μm EVO C18 100 Å LC column (100 × 2.1 mm) using 0.1% formic acid in water (v:v) as mobile phase A and 0.1% formic acid in methanol (v:v) as mobile phase B. The identification was done by comparing the transitions and retention time with reference standards. Data analysis was performed on a LabSolutions LCMS 5.6 (Shimadzu Corporation, Japan).

### DNA Extraction

Fermented samples from three time points (day 6, after stabilization and before dietary interventions; day 23, after two-week dietary interventions; and day 42, after two-week wash-out) were selected to extract bacterial genomic DNA from the pellet according to the manufacturer’s instruction of DNeasy PowerSoil Kit (12888-50, Qiagen). DNA quality and quantity were measured by a Qubit dsDNA BR Assay Kit (Q32853, Invitrogen) using a Qubit 4 Fluorometer (Thermo Fisher Scientific, USA) and then stored at −80 °C for further analysis.

### 16S rRNA Amplicon Sequencing

Bacterial genomic DNA samples were sent to Novogene Europe (Cambridge, UK) for library preparation and sequencing. Briefly, the V3-V4 region of the 16S rRNA gene was PCR amplified using 341F (5′-CCTAYGGGRBGCASCAG-3′) and 806R (5′-GGA CTACNNGGGTATCTAAT-3′) primers connected with barcodes. The PCR products were purified and then sequenced on a paired-end Illumina platform (NovaSeq 6000, Illumina) to generate 250 bp paired-end raw reads. The primers were trimmed with cutadapt 2.3.^35^ Amplicon sequence variants (ASVs) were created using DADA2,^36^ and the taxonomic assignment was performed using SILVA database v138.^37^ ASVs with the taxonomic assignment as a eukaryote, mitochondria, and chloroplast were excluded.

### Shotgun Metagenomics

Bacterial genomic DNA samples extracted from the pellet of day 23 (after dietary intervention) were selected in shotgun metagenomic sequencing performed by Novogene Europe (Cambridge, UK). Briefly, the genomic DNA was randomly sheared into short fragments, and the obtained fragments were end-repaired, A-tailed, and further ligated with an Illumina adapter. The fragments with adapters were PCR amplified, size selected, and purified. The library was checked with Qubit and real-time PCR for quantification and a bioanalyzer for size distribution detection. Quantified libraries were pooled and sequenced on NovaSeq 6000 for 4 Gb raw data per sample. The original metagenomic sequencing data were analyzed with ATLAS on default settings to harvest the functional annotation information.^38^ The genes involved in the Kyoto Encyclopedia of Genes and Genomes (KEGG) annotation of Trp metabolism (map00380), together with other potential KEGG Orthology involved in the known pathway of microbial catabolism of Trp, were chosen for further analysis.^39−41^

### Statistical Analysis

GraphPad Prism 9.1.0 (GraphPad Software, La Jolla, CA) was used for statistical analysis unless otherwise indicated. For microbial analysis, the Shannon index for α-diversity (richness and diversity) was calculated in Python3 (https://www.python.org), and β-diversity (microbial similarity or dissimilarity) was assessed by calculating a matrix of dissimilarities using the Bray–Curtis method on the relative abundance of microbiota at the genus level and then visualized using principal coordinate analysis (PCoA) by Canoco 5.14. Correlations between gut microbiota and quantified Trp catabolites were assessed with Spearman’s correlations in Scientific Python (https://scientific-python.org), in which gut microbiota is filtered by the average abundance (>1%) and prevalence (>70%). The *p*-value was adjusted for multiple tests using the false discovery rate (FDR) correction by Benjamini and Hochberg (https://tools.carbocation.com/FDR). An FDR-corrected *p*-value (FDR-*p*) < 0.05 is considered as a significant correlation, and <0.15 is considered as a speculative correlation, warranting further investigation. Significant differences in microbial community indicators and Trp catabolites between the two colon segments and diets were determined using the Student’s *t*-test. Statistical significance is represented as *p < 0.05, **p < 0.01, ***p < 0.001, and ****p < 0.0001. Sample size and statistical tests are also indicated in the figure caption.

## Results

### Microbiota Composition Varies between PC and DC and between HF and HP Diets

**Figure 2:**
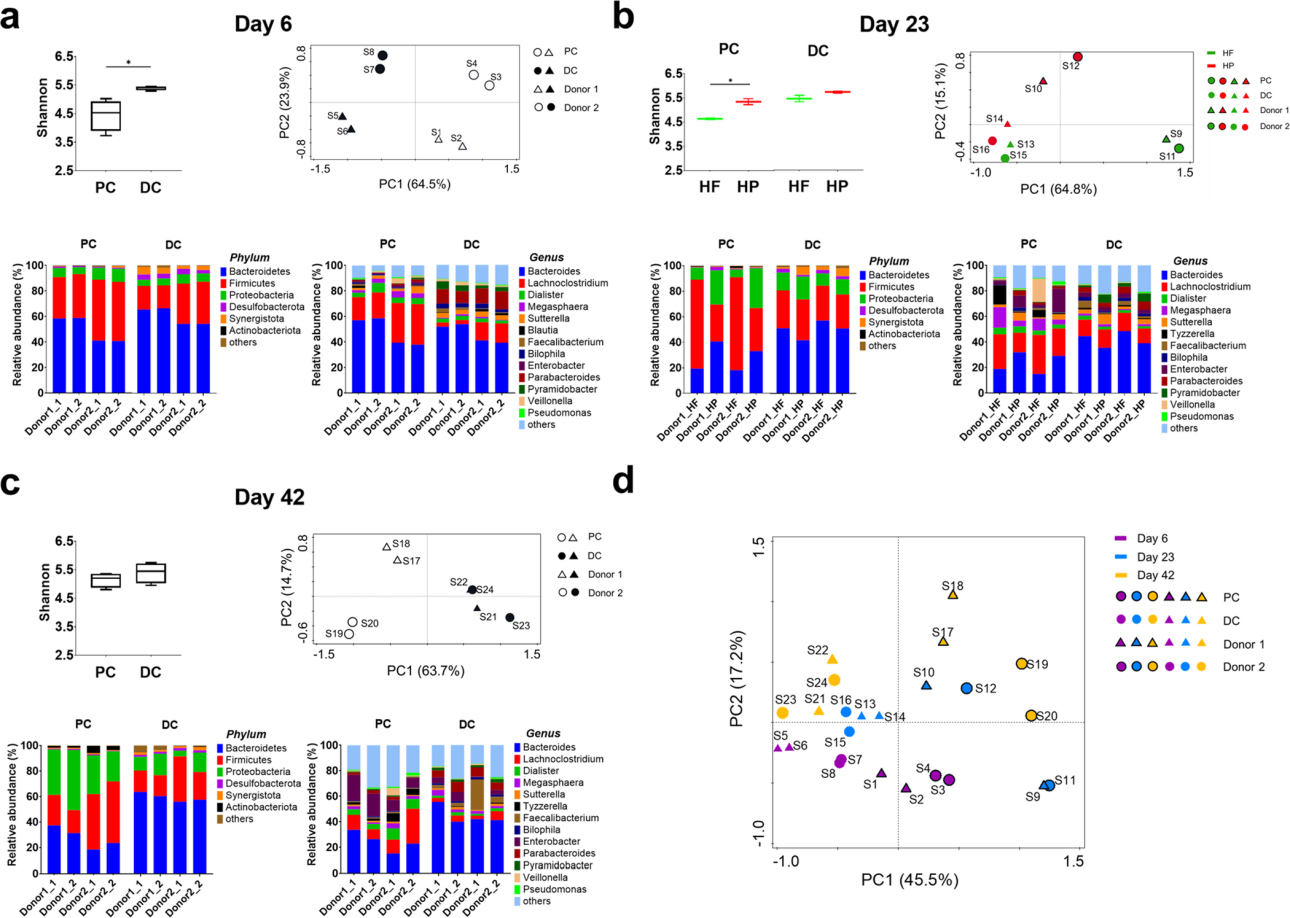
SHIME microbiota in the proximal and distal colon compartments at selected time points. In each panel, from top left to bottom right, alpha diversity (Shannon at genus level), beta diversity (PCoA with Bray–Curtis dissimilarity at genus level), and taxonomy (phylum and genus) of proximal colon (PC) and distal colon(DC) microbiota were plotted. (a) Day 6, after stabilization and before dietary interventions. (b**)** Day 23, after two-week dietary interventions by high-fiber-low-protein diet (HF, green) and high-protein-low-fiber diet (HP, red). (c) Day 42, after 2-week wash-out. (d) PCoA plot with Bray–Curtis dissimilarity of all microbiota compositional profiles at genus level. The data were obtained from two biological donors and analyzed by Student’s t test. Significance is reported as *p < 0.05. In PCoA plots, each point represents one observation (S1–S24). Donor 1 is represented by triangle and Donor 2 is represented by circle. Open symbols or symbols with black outline are PC samples. Black closed symbols or symbols without black outline are DC samples.

After stabilization time in SHIME, we compared the diversity and composition of PC and DC microbiota. We compared samples on day 7 but the initial quality control of the sequencing results suggested a contamination and thus the day 6 samples were used. The results showed that PC microbiota had a significantly lower Shannon index of α-diversity than DC microbiota (Figure 2a). The PCoA plot of β-diversity, a measure of compositional similarity, revealed differences between PC and DC microbiota, as well as between donor 1 and donor 2 (Figure 2a). To further understand these microbial dissimilarities, we evaluated their taxonomic compositions. At the phylum level, the microbial community was dominated by Bacteroidetes, Firmicutes, and Proteobacteria. PC microbiota had a higher abundance of Firmicutes and Proteobacteria, but a lower abundance of Bacteroidetes, Desulfobacterota, and Synergistota than DC microbiota. At the genus level, *Lachnoclostridium*, *Dialister*, and *Megasphaera* were more abundant in PC microbiota than in DC microbiota, whereas DC microbiota had a higher abundance of *Parabacteroides* and *Pyramidobacter* than PC microbiota (Figure 2a).

To understand how changes in the diet influence PC and DC microbiota, we conducted a two-week dietary intervention by HF and HP diets. PC microbiota exposed to HF diet had a significantly lower α-diversity than that exposed to HP diet (Figure 2b). Similarly, the microbiota β-diversity varied between HF and HP diets, especially for PC microbiota (Figure 2b). A detailed investigation of diet effect on the composition of PC microbiota revealed that the HF diet resulted in a prevalence of Firmicutes including several genera, such as *Lachnoclostridium*, *Megasphaera*, *Tyzzerella*, and *Veillonella*, whereas HP diet resulted in a prevalence of Proteobacteria represented by the genus *Enterobacter* (Figure 2b). The relative abundance of Bacteroidetes dominated by the genus *Bacteroides* was less in PC microbiota exposed to the HF diet rather than the HP diet, but this was reversed for DC microbiota (Figure 2b).

After a two-week dietary exposure and a further period of 5 days with Trp supplementation, the SHIME feed was switched to the basal diet to see if the microbiota might revert to the pre-intervention state or not. At the end of the experiment, PC microbiota from the different donors cluster together in a PCoA plot of Bray–Curtis dissimilarity at the genus level, whereas there was more variability in the biological replicates of DC microbiota from donor 1 and donor 2 (Figure 2c). An increased abundance of the phylum Proteobacteria and the genus *Enterobacter* was observed in PC microbiota compared to the pre-intervention state at day 6 (Figure 2c). Similar increases in the phylum Proteobacteria were also observed in DC microbiota (Figure 2c). These results suggest that the two-week wash-out after a dietary intervention cannot reverse the effects of the dietary modulation on the microbiota, which is further confirmed in the PCoA plot of microbiota Bray–Curtis dissimilarity of all samples (Figure 2d).

### Colon Segment Differs in the Microbial Catabolism of Trp

**Figure 3:**
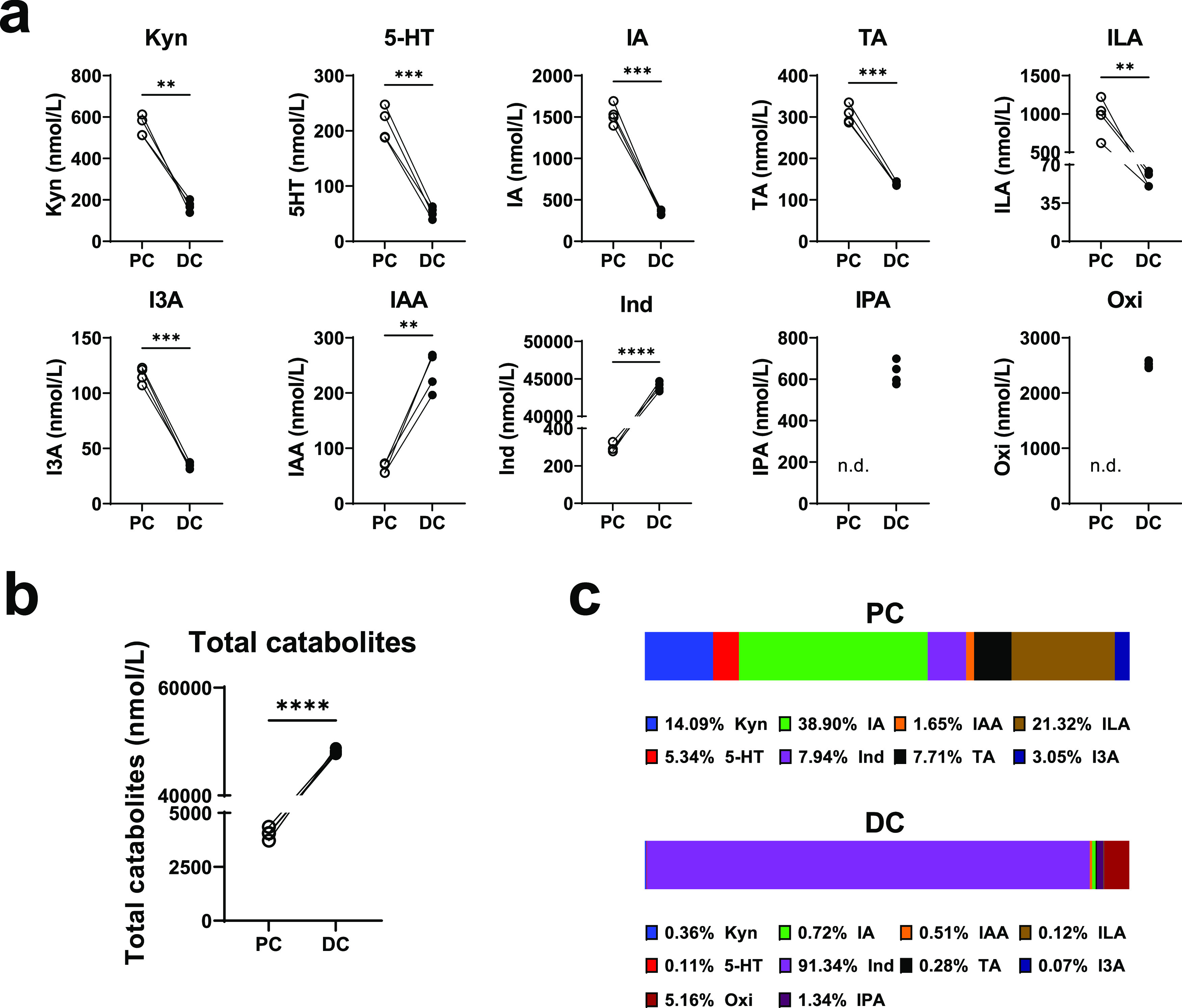
The difference in microbial production of tryptophan catabolites between proximal and distal colon. (a) Concentration of individual tryptophan metabolites (kynurenine, Kyn; serotonin, 5-HT; indoleacrylic acid, IA; tryptamine, TA; indole-3-lactic acid, ILA; indole-3- aldehyde, I3A; indole, Ind; indole-3-acetic acid, IAA; indole-3-propionic acid, IPA; oxindole, Oxi) in the proximal colon (PC) and distal colon(DC) compartments of SHIME. (b) Overall concentration of all identified catabolites in the PC and DC compartments of SHIME. (c) The average percent abundance of each catabolite relative to total identified catabolites in the PC and DC compartments of SHIME. The data were obtained from two biological donors in duplicate at Day 6 and analyzed by paired Student’s *t* test. Significance is reported as **p < 0.01, ***p < 0.001, and ****p < 0.0001.

We next examined the microbial production of Trp catabolites in the PC and DC compartments of SHIME. In line with microbiota composition, notable differences in the concentration of Trp catabolites were observed between PC and DC samples at day 6, with Kyn, 5-HT, IA, TA, ILA, and I3A at a higher concentration in PC samples than in DC samples, whereas IAA and especially Ind were more abundant in DC samples than in PC samples (Figure 3a). IPA and Oxi were only measurable in DC samples (Figure 3a).

As indicated by the overall concentration of Trp catabolites (Figure 3b), the DC is the main site for microbial catabolism of Trp and it is dominated by the production of Ind which accounts for over 90% of the measured catabolites (Figure 3c). The panel of Trp catabolites in the PC samples is completely different to DC samples, which was more balanced with IA (38.90%), ILA (21.32%), and Kyn (14.09%) being the main catabolites (Figure 3c).

### HF and HP Diets Differentially Shift the Microbial Catabolism of Trp

**Figure 4:**
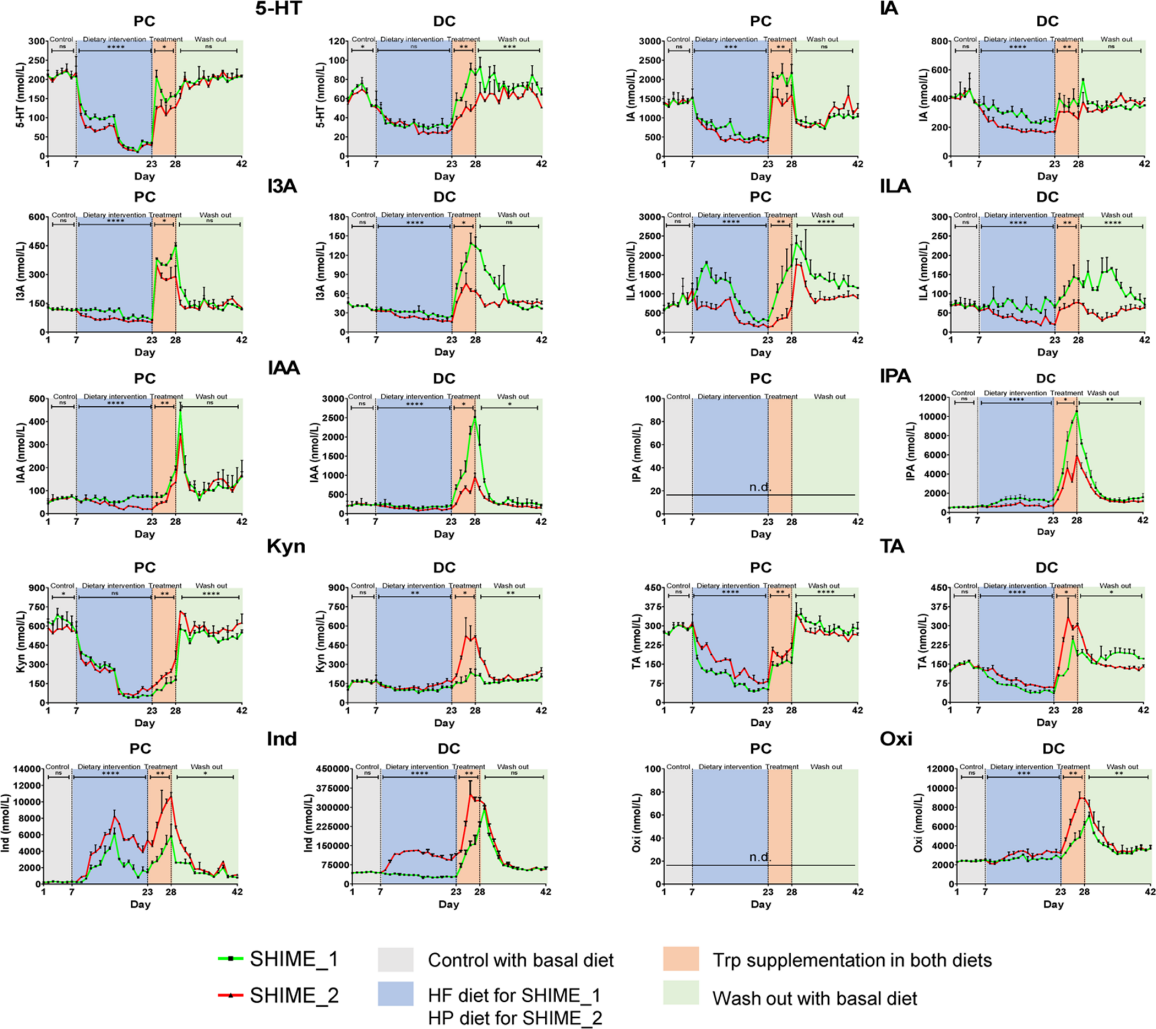
Microbial production of tryptophan catabolites during the experimental period. Control (gray): SHIME_1 and SHIME_2 were fed with basal diet. Dietary intervention (blue): SHIME_1 was fed with HF diet and SHIME_2 was fed with HP diet. Treatment (orange): supplied 0.2 g/L tryptophan in the HF/HP diet. Wash out (green): SHIME_1 and SHIME_2 were fed with basal diet. n.d.: not detected. The data are presented as mean + SD (*n* = two biological donors) and analyzed by paired Student’s t test between SHIME_1 and SHIME_2 in each period. Significance is reported as ns *p* > 0.05, *p < 0.05, **p < 0.01, ***p < 0.001, and ****p < 0.0001. 5-HT, serotonin; IA, indoleacrylic acid; IAA, indole-3-acetic acid; ILA, indole-3-lactic acid; I3A, indole-3-aldehyde; IPA, indole-3-propionic acid; Kyn, kynurenine; TA, tryptamine; Ind, indole; Oxi, oxindole. PC: proximal colon. DC: distal colon.

To understand how changes in the diet influence the microbial production of Trp catabolites, we conducted a two-week dietary intervention by HF and HP diets in SHIME. After both dietary interventions, a rapid decrease in the microbial production of 5-HT, IA, and TA was observed in both PC and DC samples and Kyn in PC samples, whereas Ind rapidly increased and then decreased in PC samples (Figure 4). Trp catabolism by gut microbiota rapidly changed in response to the different diets. The HF diet resulted in a higher concentration of 5-HT, IA, IAA, ILA, I3A, and IPA than the HP diet in both PC and DC samples, whereas the HP diet resulted in a higher concentration of Ind, TA, Kyn, and Oxi than the HF diet (Figure 4).

To verify the Trp catabolizing ability of the microbiota adapted to different diets, we supplied 0.2 g/L free Trp in HF or HP diet at the end of the dietary intervention for 5 days. As expected, Trp supplementation increased the microbial production of Trp catabolites in both PC and DC samples (Figure 4). The microbiota adapted to HF diet maintained a stronger capacity to produce 5-HT, IA, IAA, ILA, I3A, and IPA than the microbiota adapted to HP diet, whereas the microbiota adapted to HP diet maintained a stronger capacity to produce TA, Kyn, Ind, and Oxi than the microbiota adapted to the HF diet (Figure 4).

To examine whether dietary interventions have a lasting effect on Trp catabolism by gut microbiota, we washed out the SHIME diet with the basal diet for two weeks. After the wash-out period, the microbial production of most of Trp catabolites in PC and DC samples returned to the pre-intervention state (Figure 4). However, the microbiota adapted to the HF diet retained a strong capacity to produce ILA and IPA and the microbiota adapted to the HP diet retained a strong capacity to produce Kyn (Figure 4). Furthermore, the microbiota adapted to the HF diet had a stronger capacity to produce TA than the microbiota adapted to the HP diet, which was different to what was observed in the dietary intervention (Figure 4).

### Diet Modulates the Genetic Potential of the Microbiota To Produce Trp Catabolites

**Figure 5:**
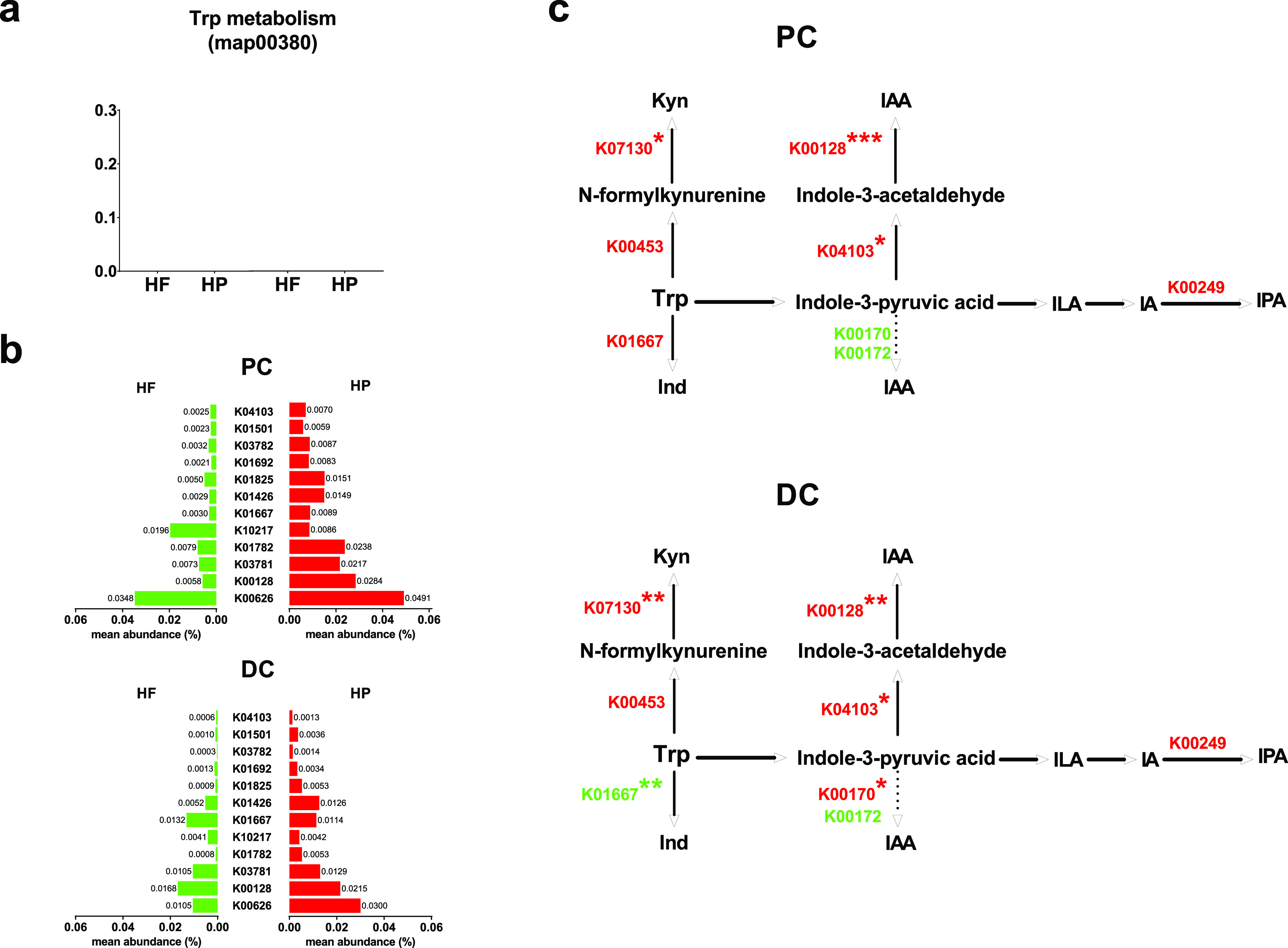
Differential functions of the microbiome regarding tryptophan catabolism between HF and HP diets. (a) The comparison of microbial Kyoto Encyclopedia of Genes and Genomes (KEGG) pathway of tryptophan (Trp) metabolism (map00380) between proximal colon (PC) and distal colon(DC) microbiota exposed to high-fiber-low-protein (HF, green) diet and high-protein-low-fiber (HP, red). (b) The mean abundance of the top 12 abundant KEGG Orthology (KO) in the map00380. (c) Cartoon displays the Trp catabolism to kynurenine (Kyn), indole (Ind), indole-3-acetic acid (IAA), and indole-3-propionic acid (IPA) and the KO involved in each pathway and identified in the PC and DC microbiota exposed to HF and HP diets. The color represents the relatively high abundance of KO in which diet with significance marked by asterisks. The details are given in Figure S1. The data were obtained from two biological donors at Day 23 and analyzed by Student’s *t* test. Significance is reported as *p < 0.05, **p < 0.01, and ***p < 0.001. ILA: indole-3-lactic acid; IA: indoleacrylic acid.

We next analyzed metagenomic sequencing data at day 23 to investigate the diet effect on the functional profiling of the microbiome regarding Trp catabolism. The selected genetic potential of PC and DC microbiota to produce Trp metabolites was assessed by determining the normalized relative abundance of DNA sequence reads mapping to the superpathway of KEGG Trp metabolism (map00380). Genes involved in Trp metabolism were relatively more abundant in the PC microbiota than in DC microbiota (Figure 5a), and nearly all the abundant KEGG Orthology (KO) in the map00380 was enriched by the HP diet, with the exception of K10217 in PC microbiota and K01667 in DC microbiota (Figure 5b). A search was performed for all known enzymes or KOs involved in the microbial catabolism of Trp.^39−41^ A total of six relevant KOs were found, as well as two candidate genes (K00170 and K00172) responsible for the conversion of indole-3-pyruvic acid to IAA (Figure 5c).^21^ The HP diet enriched the KOs involved in the biosynthesis of Kyn (K00453 and K07130), IAA (K04103 and K00128), and IPA (K00249) in both PC and DC microbiota, whereas the HF diet enriched the K00172 (Figures 5c and S1). Interestingly, the effect of diet on the abundance of K01667 and K00170 varied between the PC and DC microbiota (Figures 5c and S1).

### Correlation between the Gut Microbiota and Trp Catabolites

**Figure 6:**
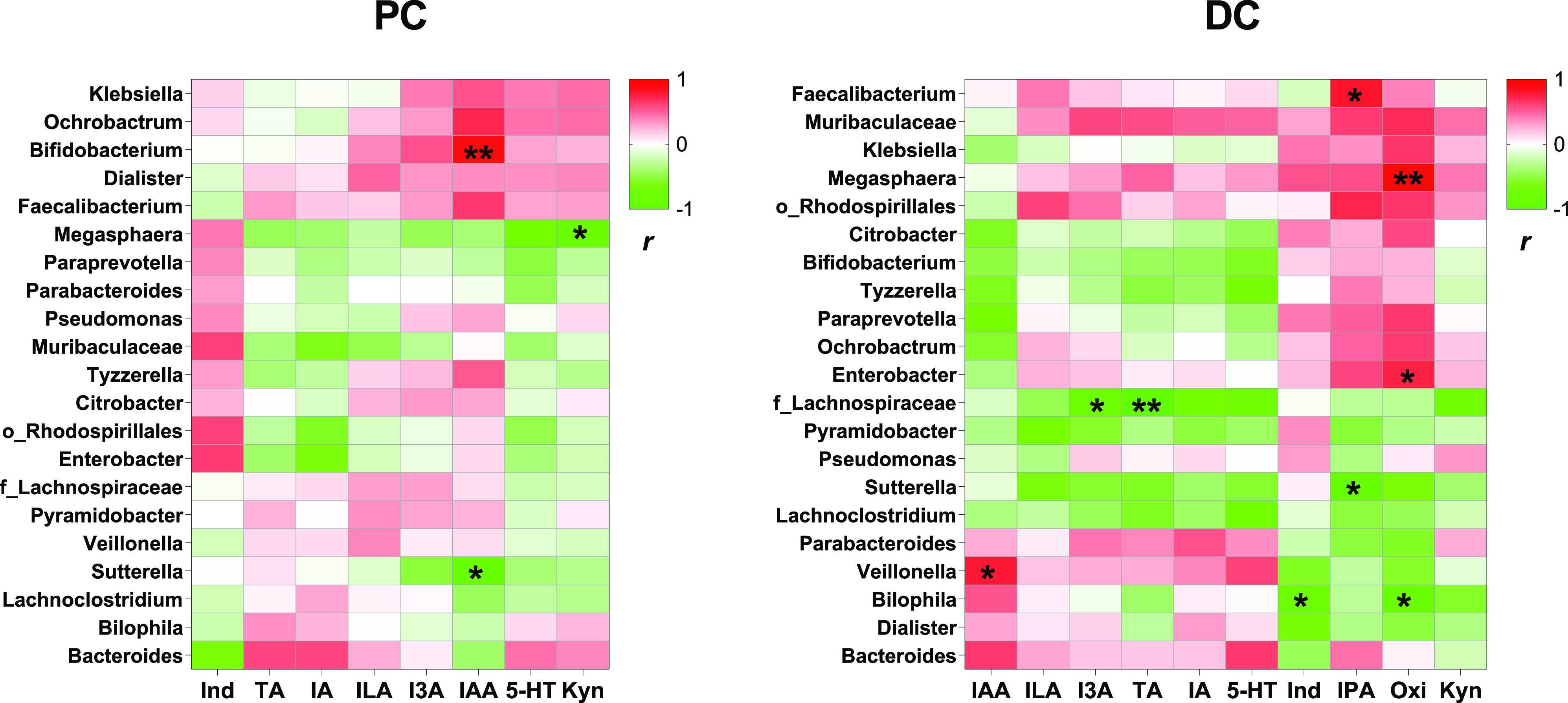
Correlations between gut microbiota and tryptophan catabolites. The microbial genera with a mean relative abundance >1% and represented in >70% of proximal colon (PC) or distal colon (DC) microbial samples obtained from Day 6, Day 23, and Day 42 were correlated to their corresponding quantified tryptophan catabolites through non-parametric Spearman rank analysis. The heatmap was plotted based on *r* values. The red color means positive correlation and the green color means negative correlation. **Significant correlation with FDR-corrected *p* value <0.05. *Potential correlation with FDR-corrected *p* value <0.15. Kyn: kynurenine; 5-HT: serotonin; IA: indoleacrylic acid; Ind: indole; IAA: indole-3-acetic acid; TA: tryptamine; ILA: indole-3-lactic acid; I3A: indole-3-aldehyde. Oxi: oxindole; IPA: indole-3-propionic acid.

We further identified the correlation between the gut microbiota and Trp catabolites. The microbial genera that made up less than 1% of the total counts and occurring in less than 70% of samples were removed from the analysis. Overall, although the correlations were relatively weak after the FDR correction, significant differences in the correlation patterns of PC and DC microbiota for Trp catabolites were observed, for example *Megasphaera* in PC microbiota was negatively correlated with most of Trp catabolites produced in the PC compartment of SHIME, but positive correlations between *Megasphaera* and most of Trp catabolites were observed in DC samples (Figure 6). In PC samples, a significantly positive correlation was observed between *Bifidobacterium* and IAA (FDR-*p* < 0.05), and potentially negative correlations were observed between *Megasphaera* and Kyn and between *Sutterella* and IAA (FDR-*p* < 0.15) (Figure 6). In DC samples, *Megasphaera* was positively correlated with Oxi (FDR-*p* < 0.05), as well as *Enterobacter* (FDR-*p* < 0.15); *Faecalibacterium* was positively correlated with IPA (FDR-*p* < 0.15); *Veillonella* was positively correlated with IAA (FDR-*p* < 0.15); uncultured_f_*Lachnospiraceae* was negatively correlated with I3A (FDR-*p* < 0.15) and TA (FDR-*p* < 0.05); *Sutterella* was negatively correlated with IPA (FDR-*p* < 0.15); and *Bilophila* was negatively correlated with Ind and Oxi (FDR-*p* < 0.15) (Figure 6).

## Discussion

Given the beneficial role of microbiota-derived Trp catabolites in intestinal homeostasis and immune regulation,^10,42,43^ we investigated how the HF or HP diet influences the capacity of human gut microbiota to produce Trp catabolites in SHIME. We found that the composition and function of human gut microbiota was different in the proximal and distal colon compartments. After the *ex vivo* human gut microbiota was stabilized in SHIME, DC microbiota showed a higher diversity than PC microbiota, consistent with a previous study in humans.^44^ The major intestinal phyla Bacteroidetes and Firmicutes were present in both PC and DC microbiota but Bacteroidetes were more abundant in the DC microbiota (∼60% versus 50%), and Firmicutes were more abundant in PC microbiota (∼40% versus 25%). The acidic pH of 5.6–5.9 in the PC compartment of SHIME provides a competitive advantage for acid-tolerant bacterial species, mostly from Firmicutes,^45^ to grow in this niche, whereas members of Bacteroidetes grow poorly in acidic conditions and grow optimally in the DC compartment, where the pH was maintained at 6.6–6.9.^46^ The substrates available for metabolism also influence the microbiota composition. In the colon, the microbiota preferentially utilizes fermentable carbohydrates over proteins for growth.^47^ Consequently, the proximal colon is the main site for saccharolytic fermentation. When fermentable carbohydrates are depleted, the microbiota switch to other energy sources, such as peptides or amino acids, and this typically occurs in the distal colon.^48^ Firmicutes consist of many degraders of complex carbohydrates, although Bacteroidetes, mostly *Bacteroides* spp., have the largest repertoire of carbohydrate-active enzymes of all intestinal microbiota.^49^ However, *Bacteroides* spp. are usually considered as generalists because of their capacity to utilize a large range of substrates for growth including proteins.^46^

The microbial production of Trp catabolites is not limited to specialized protein degraders or to the distal colon. Several Trp catabolites, such as Kyn, 5-HT, IA, TA, ILA, and I3A, were quantified at high concentrations in PC samples. This may be due to the high abundance of acid-tolerant bacteria in PC microbiota,^46^ e.g., *Bifidobacterium* spp. (Figure S2), which is capable of converting Trp into ILA.^50^ Kyn and 5-HT are two well-known endogenous Trp metabolites, but a growing body of literature suggests that they can also be produced by gut microbiota.^4,15,24,41,51^ Our data further support this notion. We identified the enzymes responsible for Kyn biosynthesis, Trp 2,3-dioxygenase (K00453) and arylformamidase (K07130),^40^ which were more abundant in PC microbiota than in DC microbiota (Figure S1) but not the enzymes for microbial biosynthesis of 5-HT.^41^

Large amounts of Trp catabolites were identified in the DC samples, especially Ind via the activity of tryptophanase (K01667) in line with the distal colon being the most active location for proteolytic fermentation of proteins.^46,52^ Within the human gut microbiota, tryptophanase is widely expressed in the commensal *Bacteroides* species,^23^ which are suggested to be active in the distal colon and inactive in the proximal colon because of the environmental pH.^53^ Several *Bacteroides* species have also been reported to produce IAA.^54^

One stark difference in Trp catabolism between PC and DC microbiota was the production of IPA and Oxi, which was only identified in DC samples. IPA has recently received increased attention for its association with gut barrier integrity and cognitive performance,^12,19^ as well as its protective role in disease.^55,56^ To date, only a small number of bacteria, mostly *Clostridium* spp.,^21,57^ have been identified for IPA production. Very little information is available on the microbial production of Oxi. A recent study indicated that Oxi is present at a considerable concentration in human stool, and it can activate human AhR at physiologically relevant concentrations.^24^ However, the physiological significance of Oxi, and the identity of the bacteria producing it in the human gut, are currently unknown. Our correlation analyses suggest that *Megasphaera* and *Enterobacter* might be the main contributors to Oxi production in the distal colon.

In addition to intestinal location-specific differences, we also demonstrated the diet-induced modifications to microbiota composition and microbiota-derived Trp catabolites. Changes in microbial community reflect trade-offs between primary utilization of fermentable carbohydrates versus proteins in the diet. A study on professional athletes revealed that athletes have a high diversity of gut microbiota and this is positively correlated with the high level of protein consumption.^58^ We also found that the HP diet increased the diversity of PC and DC microbiota compared to HF diet. A distinct diet effect on microbiota composition was observed in PC microbiota: the HF diet favored fiber-degrading bacteria, especially those from Firmicutes, whereas the HP diet allowed the increase of acid-sensitive Proteobacteria.^59^ Surprisingly, a similar effect was not observed in DC microbiota. One possible explanation is the high abundance of *Bacteroides* in DC microbiota, which is able to cope with the different diets using its large repertoire of degrading enzymes.^46^

The modification of microbiota composition induced by the different diets has been previously associated with changes in Trp catabolism. For example, the 4-day Mediterranean diet has been reported to increase the concentration of IAA, IPA, and ILA in the plasma of individuals.^25^ Our data demonstrated that, compared to a HP diet, the HF diet favored the microbial production of 5-HT, IA, IAA, ILA, I3A, and IPA. This suggests that some of the fiber-degrading bacteria enriched by HF diet may have the capacity to specifically catabolize Trp. The HF diet has a lower content of protein compared to the HP diet and therefore a lower content of Trp precursors. Thus, we speculate that the effect of fiber on microbial catabolism of Trp is independent of protein or Trp content, but largely dependent on the effect of the diet on the abundance of microbes able to catabolize Trp.

Compared to a HF diet, the HP diet favored the microbial production of TA, Kyn, Ind, and Oxi, but this may be due to the higher content of Trp in the HP diet. Thus, we analyzed the functional capacity of the microbiota to produce beneficial Trp catabolites by shotgun metagenomic sequencing. The HP diet increased the genetic potential of PC and DC microbiota for Kyn biosynthesis compared to the HF diet, as well as the PC microbiota for Ind biosynthesis. Interestingly, the HP diet reduced the genetic potential of DC microbiota for Ind biosynthesis, which was not in agreement with the higher concentration of Ind in DC samples after the HP dietary intervention. Similar inconsistencies were also observed for IAA and IPA production and measured gene abundances. This may be due to the fact that the identified Trp catabolic genes are not all expressed in PC and DC microbiota.^60^

The diet effect on gut microbiome is rapid and reproducible.^61^ Previous trials in humans showed gut microbiota can recover from dietary interventions to its pre-intervention baseline state after a wash-out period of 3–4 weeks.^62,63^ However, an intensive wash-out of 2 weeks in SHIME was unable to reverse the diet effect on PC and DC microbiota, and even resulted in a different microbial community compared to the pre-intervention state. A recent study demonstrated that the rapid evolution of the gut microbiota occurs in the response to dietary change influencing the microbiota-dependent phenotypes in humans.^64^

SHIME allows a good representation of the in vivo gut microbial communities and offers unique advantages in studying location-specific differences in human gut microbiome and long-term dietary intervention, but it can only be performed with a limited number of donors. This limits statistical power and the comparison of statistical significances in the results. Thus studies with different donors would be needed to translate the findings to a broader group of individuals. To increase the confidence in our findings, we longitudinally measured the catabolite data and run the SHIME for a long period of time (∼8 weeks), but to completely understand the complexity of the Trp catabolism by gut microbiota, more in-depth metagenomic and metatranscriptomic analysis need to be performed on a large number of human fecal donors.

Despite the limitations, this study presents the first detailed observation of the location-specific differences in microbiota composition and microbiota-associated Trp catabolism of the human PC and DC microbiota, and provides the first detailed characterization of the shifts in microbial catabolism of Trp under contrasting diets differing in the relative abundance of fiber and proteins. This study establishes the bacterial genera that are likely to be the main contributors to the microbial catabolism of Trp in the human colon. The results of this study increase the understanding of tryptophan catabolism by gut microbiota along the colon and will contribute to the design future strategies based on dietary interventions to favor microbial production of beneficial Trp catabolites.

## Supporting Information Available

Comparison of the specific KEGG Orthology in the identified tryptophan catabolism pathway (Figure S1) and the relative abundance of Bifidobacterium in proximal colon (PC) and distal colon(DC) microbiota (Figure S2) (PDF)

## Abbreviations Used

5-HT: serotonin
AhR: aryl hydrocarbon receptor
DC: distal colon
I3A: indole-3-aldehyde
IA: indoleacrylic acid
IAA: indole-3-acetic acid
ILA: indole-3-lactic acid
Ind: indole
IPA: indole-3-propionic acid
Kyn: kynurenine
Oxi: oxindole
PC: proximal colon
SHIME: simulator of human intestinal microbial ecosystem
SCFA: short-chain fatty acid
TA: tryptamine
Trp: tryptophan
