# Supplementary material for: Impact of High-Fiber or High-Protein Diet on the Capacity of Human Gut Microbiota To Produce Tryptophan Catabolites
Source: J Agric Food Chem. 2023 May 1;71(18):6956–66. doi: 10.1021/acs.jafc.2c08953 (PMC10176579; doi:10.1021/acs.jafc.2c08953)
Supplement: Supplementary file 1 — jf2c08953_si_001.pdf [file jf2c08953_si_001.pdf]

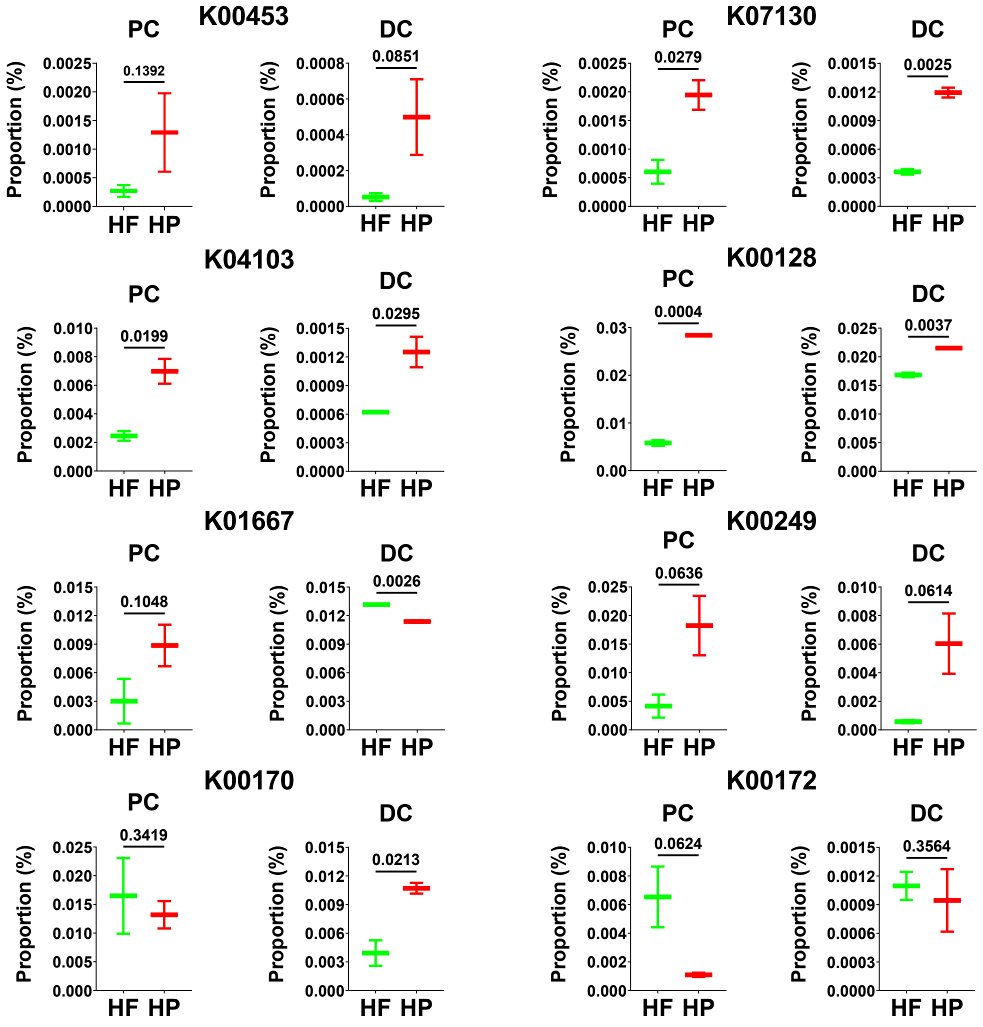

Figure S1 Comparison of the specific KEGG Orthology in the identified tryptophan catabolism pathway. PC: proximal colon. DC: distal colon. HF: high-fiber-low-protein diet. HP: high-protein-low-fiber diet. Data were from two biological donors at Day 23 and were analysed by Student's t test. The *p* value is shown in each plot and lower than 0.05 is considered as significant difference.

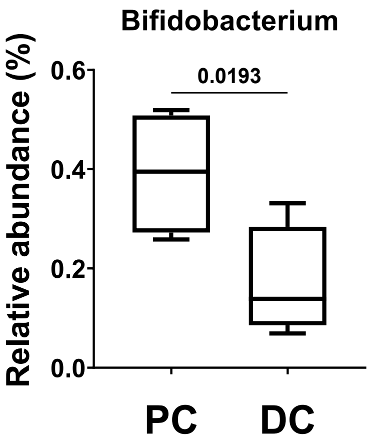

Figure S2 Relative abundance of *Bifidobacterium* in proximal colon (PC) and distal colon (DC) microbiota. Data were from two biological donors in duplicate at Day 6 and analysed by Student's t test. The *p* value is shown in the plot and lower than 0.05 is considered as significant difference.
